# Patient Evaluation of Food Waste in Three Hospitals in Southern Italy

**DOI:** 10.3390/ijerph16224330

**Published:** 2019-11-06

**Authors:** Sara Schiavone, Concetta Paola Pelullo, Francesco Attena

**Affiliations:** Department of Experimental Medicine, University of Campania “Luigi Vanvitelli”, 80138 Naples, Italy; sara.schiavone@unicampania.it (S.S.); concettapaola.pelullo@unicampania.it (C.P.P.)

**Keywords:** food waste, hospital, foodservice, plate waste, hospitalized patients

## Abstract

In recent years, food waste has received great attention and is now considered the cause of many negative effects, including health, economic, social and environmental issues. A cross-sectional study was conducted among a sample of 762 inpatients at three hospitals of Campania region in Italy. The purpose of this study was to evaluate the amount of food waste occurring in these hospitals using a structured questionnaire and asking inpatients about the average percentage of food they had disposed of in the previous three days. The overall food wasted amounted to 41.6%. The main plates, first (pasta or rice), second plate (meat or fish), resulted in similar amounts of waste (38.5% and 39.7%, respectively). The side plate (vegetable or potatoes), however, generated the greatest amount of waste (55.0%); 40.7% of patients totally discarded this part of their meals. The type of food wastage among the three hospitals reflected similar patient behaviours, with the amount of food wasted never falling below 30%. Females tended to waste more food than males (59.1% vs. 38.2%; *p* = 0.000). Other variables were correlated with less food waste, such as having a good opinion of the food’s quality (RR = 1.91; 95% C.I. = 1.68–2.17) and satisfaction with the foodservice in general (RR = 1.86; 95% C.I. = 1.64–2.10). Poor quality, different eating habits and the feeling of satiety were the main reasons patients gave for food waste. Our study suggests that the most promising way to reduce food waste in hospitals is to improve the quality of meals and to establish an individual, simplified and flexible meal reservation process based on specific needs and preferences.

## 1. Introduction

Food waste is defined by the Food and Agricultural Organization (FAO) as food appropriate for human consumption being discarded, whether after it is left to spoil or kept beyond its expiry date [1]. In recent years, food waste has received great attention and is now considered the cause of many negative effects, including health, economic, social and environmental issues [2]; it is estimated to cost 1000 billion a year [3]. According to the United Nations Food and Agriculture Organization, 1.3 billion tons of food is lost or wasted around the globe each year, which amounts to 1/3 of all food produced for human consumption. The global quantities of food lost or wasted by commodity group can be disaggregated into the following percentages: fruit and vegetables, 45%; roots and tubers, 45%; fish and seafood, 35%; cereal, 30%; dairy products, 20%; oilseeds and pulses, 20%; and meat, 20% [4].

Food is lost or wasted throughout the food supply chain: on the farm, in processing and manufacturing, in shops, and in restaurants, hospitals and homes. Factors contributing to food waste include insufficient shopping and meal planning, leading to too much food being purchased or prepared; misunderstandings about the meaning of ‘best before’ and ‘use by’ date labels leading to edible foods being thrown away; standardised portion sizes in community facilities (e.g., schools, hospitals); difficulty in anticipating the number of customers; inadequate storage or transport at all stages of the food chain; and poor-quality food [5,6,7]. A FAO report focused on the global impacts of food wastage (i.e., both food loss and food waste) on the environment and on natural resources along the food supply chain, particularly on climate, water, land and biodiversity [1]. From this point of view, food waste is considered an important indicator of sustainability because it represents all the resources used to produce uneaten food, including cropland, agricultural chemicals such as fertilizers and pesticides, and irrigation water; in other words, all these resources are exploited to grow food that is ultimately wasted by consumers [8].

In Italy, the care activities in hospital are regulated by the DRG system. DRGs offer a framework for an accurate assessment of the costs of treating a given patient. In a DRG system, each meal has a price that takes resources away from the rest, such as treatment, diagnostics and assistance. In this perspective, food waste can affect the whole outcome of the hospitalized patient [9]. Therefore, in hospitals, food waste takes away funding from general patient treatment. Many initiatives have been undertaken to reduce such waste. The Italian Government [10] promulgated a law titled ‘Provisions Concerning the Donation and Distribution of Food and Pharmaceutical Products for Purposes of Social Solidarity and for the Limitation of Waste’, which encourages the recovery of food through charitable donations. In a project titled ‘Causes of Food Waste and Corrective Actions’, the Italian Ministry of Health [11] involved students to promote correct lifestyles and to stem the specific phenomenon of food waste. The Ministry later promoted a collaboration with public institutions (such as schools, hospitals and public companies) titled ‘Guidelines Addressed to the Managing Bodies of School, Company, Hospital, Social and Community Canteens, in Order to Prevent and Reduce Food-related Waste’ [12].

The aim of our study was to estimate the amount of food wasted by inpatients and to evaluate the causes of this discard. The most common method used to evaluate food waste in hospitals is plate waste, which refers to the percentage of the served food that is discarded. Generally, plate waste is measured in two ways: by weighing the food left on the plate or by visual estimation. In our study, we tested another method. We interviewed patients in three hospitals of Campania region in Italy using a structured questionnaire and asked them about the average percentage of food they had disposed of in the last three days.

## 2. Materials and Methods

### 2.1. Setting

This cross-sectional study used data provided by inpatients at three hospitals of Campania region in Italy from March to November 2018. These three hospitals have similar characteristics: they are highly specialised (in Italy such facilities are called *aziende ospedaliere*), they outsource their kitchen services and they use a plate meal delivery system to serve lunch and dinner. The food for each ward is loaded into food containers and transported from a central kitchen. Meals are served three times a day: breakfast is delivered from 07.30 to 08.30, lunch from 12.00 to 13.00 and dinner from 18.00 to 19.00. Food is ordered by the nurse responsible for each ward based on the number of inpatients and the medical prescriptions listed on patients’ clinical charts (standard meals or modified meals for specific needs such as dysphagia, diabetes, high energy, high protein, etc.). In the selected hospitals, and generally, in Italy, lunch and dinner usually comprise three separate plates called ‘first’, ‘second’, and ‘side plate’ (contorno in Italian), as well as an additional fruit. The first plate is usually pasta or rice and is seasoned with tomatoes, legumes or vegetables, or includes both. The second plate consists of cooked meat or fish. The side plate, served on a separate plate, consists of potatoes or other legumes or vegetables. The accompanying fruit is usually an orange, peach, pear or apple. This study was carried out in different wards for each hospital, both medical and surgical, with the exclusion of the psychiatric and intensive care wards. Some features of the three hospitals are

- Hospital 1: 580 beds. The kitchen is located 10 km from the hospital; therefore, the food is prepared and kept at 65 °C for about 2 h before consumption.
- Hospital 2: 487 beds. The food is prepared in the hospital’s internal kitchen managed by an external service and distributed after 30 min at most.
- Hospital 3: 875 beds. The food is prepared in the hospital’s internal kitchen managed by an external service and distributed after 30/40 min at most.

### 2.2. Data Collection

During the study period, we included patients who had been hospitalised for at least three days. We excluded non-collaborating patients and those prescribed special diets. The patients were interviewed by two physicians trained in epidemiology and public health 1–2 days each week on different days. Participants gave their written consent to participate in the study and were informed that all data collected would be analysed in aggregate and that confidentiality would be strictly protected. Research ethics committee approval for this study was obtained from the Ethics Committee of the University of Campania “*Luigi Vanvitelli*” (prot. N. 405/2018).

### 2.3. Sample Size

The previously calculated sample size consisted of 713 patients, assuming a 35% rate of expected prevalence of the percentage of food wasted, with a margin of error of 3.5% and a level of significance of 95%.

### 2.4. Questionnaire

The participants were asked to provide the following information (Supplementary File S1: Questionnaire): (a) Sociodemographic data: age, sex, nationality, education, marital status and employment; (b) Characteristics of food served: quality, variety, presentation, quantity, trust in the safety of food, right temperature, importance placed on the meal; (c) Characteristics of foodservice: foodservice satisfaction, time of meal distribution, courtesy of the staff serving food; d) Rate of food waste (main outcome): to evaluate the amount of food discarded we used the following question: “In which percentage did you consume your meals?”. This question was asked regarding the first plate, second plate, side plate and fruit (answered using a 5-point Likert scale: nothing/almost nothing, about 1/4, about half, about 3/4, all/almost all). ‘Almost nothing’ means that patients just tasted the food and then refused it. We also asked the patients why they discarded food and whether they brought in food from home or from another external catering service.

Some questions included in the questionnaire were based on the ‘Acute Care Hospital Foodservice Patient Satisfaction Questionnaire (ACHFPSQ)’ [13], an instrument used to measure patient satisfaction with a hospital’s foodservice.

The questionnaire contained items rated on a 5-point Likert scale from “always” to “never” and an overall rating from “very good” to “very poor”, and from “none” to “very much”. It took approximately 15 min to complete the recording.

### 2.5. Measurement of Food Waste (Wastage Rate)

Starting with the question: “In which percentage did you consume your meals?”, the overall food wasted for each plate was calculated as follows: (percentage of patients who discarded 100% of their food × 1) + (percentage of patients who discarded 75% of their food × 0.75) + (percentage of patients who discarded 50% of their food × 0.50) + (percentage of patients who discarded 25% of their food × 0.25) + (percentage of patients who discarded 0% of their food × 0) (Table 1). The overall amount of food discarded was calculated as the weighted average of the three plates in relation to their average weight, given that the first plate was weighted as 1.00: first plate 1.00, second plate 0.60, side plate 0.50 and fruit 0.40. These values were calculated for the three days of food served in the three hospitals.

### 2.6. Statistical Analysis

Descriptive analyses were conducted for all the variables. Univariate analyses were performed between the main outcomes (“In which percentage did you consume your meals?”) and all the other variables of the questionnaire. The calculation of the risk ratios of all variables was dichotomised: only variables with a *p* value ≤ 0.25 were subsequently included in the multivariate logistic regression model and the adjusted *p* values were calculated. Analyses were carried out using the statistical software package SPSS Version 21.0 (IBM Corp, Armonk, NY, USA).

## 3. Results

### 3.1. Socio-Demographic Characteristics

Of the 872 inpatients approached, 110 refused to participate, giving us a response rate of 87.4%, and 762 participants. The respondents’ ages ranged between 18 and 94 years and were equally distributed among these two age groups: 18–60 years old (50.3%) and 61–94 years old (49.7%). Other demographic characteristics for this group included the following: 58.1% were women, 75.3% were married, 55.3% had a low level of education, 28.5% were unemployed (most of these individuals were homemakers), and only 4.5% were foreigners (34 people) (see Table 2).

### 3.2. Food Waste

Table 3 shows the amounts of food wasted according the statements of the inpatients of the three hospitals. Overall, 41.6% of the food served was wasted. The main plates, first plate (pasta or rice), second plate (meat or fish) and fruit, were subject to similar amounts of waste (38.5%, 39.7% and 35.2%, respectively). The plate most often wasted was the side plate (vegetable or potatoes), with 55.0% being discarded; 40.7% of patients discarded their side plates entirely. The variability of food wasted among the three hospitals showed a similar patient behaviour, with the amount of food wasted never falling below 30%.

Regarding socio-demographic characteristics (Table 4), females appeared to waste more food than males (59.1% vs. 38.2%); this was demonstrated in both the univariate and multivariate analyses (*p* = 0.000). In the univariate analysis (*p* = 0.005) the patients who were unemployed seemed to discard more food than those who were employed, but this association was not present in the multivariate analysis, probably because no distinction was made regarding gender, although fewer women were employed than men. No association was found between food waste and age, education or nationality (*p* > 0.05).

Table 5 shows patients’ opinions of various aspects of the hospitals’ food and foodservice quality, disaggregated by food wasted. In general, 82.5% of patients placed high/very high importance on mealtimes, and they reported positive opinions of all aspects of hospital catering, in terms of both food quality and foodservice, all variables of which were included between two-thirds and three-quarters of the positive answers. The more positive results concerned the courtesy of the staff (often/always = 92.3%).

Regarding the variables associated with food being discarded, in the univariate analysis, almost all the aspects of a good food and foodservice quality were associated with less food being discarded. In particular, the variables more strictly correlated with less food waste were a good opinion of the food’s quality (RR = 1.91; 95% C.I. = 1.68–2.17; *p* = 0.000) and satisfaction with the foodservice in general (RR = 1.86; 95% C.I. = 1.66–2.10; *p* = 0.000). However, a multivariate analysis which included the variables presented in Table 4 and Table 5 with *p* < 0.25 showed that only these two last variables were statistically associated with less food waste. Finally, both the univariate and multivariate analyses showed that many respondents who did not consume hospital meals ate food from home or other catering services brought to them by relatives during visiting hours (RR = 1.59; 95% C.I. = 1.31–1.92; *p* = 0.000).

The primary reasons for food waste related to the characteristics of the food served, poor quality, different eating habits, and feeling of satiety accounted for 56.4% of the reasons for food being discarded. The remaining answers were poor appetite, other reasons or no answer, including those who had consumed all of the food they had been served during the three days (Table 6).

## 4. Discussion

To our knowledge, this is the first patient survey on the subject of food waste. According to this methodology, 41.6% of the food served was wasted in the three hospitals, with the side plate being the most often discarded dish. This rate is close to the highest rates identified by other studies, both recent and older, most of which reported rates ranging between 25% and 40% [14,15,16,17,18,19,20,21,22,23]. The only Italian study conducted in several hospitals in the Piedmont region [24] reported that 31.2% of the food served was wasted. Moreover, our results do not account for the food wasted along the supply chain, the addition of which could bring the overall waste rate to well over 50%. Such a high amount of food waste could also have health effects related to reduced calorie intake and not just wasting resources. However, we would remind that this reduced intake would be compensated by the patients’ habit of bringing food from home or other catering services, which is used by up to a third of patients.

Disaggregating the data showed that the food waste rates were similar in the three hospitals, indicating that our results may reflect a general and widespread tendency to waste food in this geographic area. Unpleasant quality was the main variable statistically correlated to greater food waste, which was the result of the multivariate analysis.

The widespread habit of hospitals of wasting food has rightly provoked several reactions, suggestions and initiatives aimed at reducing it. The most obvious intervention is to improve the quality and presentation of food [5]. Other initiatives concern the way food is delivered; it has been shown that a bulk food delivery system in which a variety of food is brought to patients and served from trollies according to each patient’s appetite and choice reduces the amount of wasted food more effectively than standard plated meal delivery systems [25]. Another alternative to the standard plate delivery is the bedside menu ordering system [14,26], which McCray et al. found to decrease food waste from 30% to 26%. Similar to the bedside menu ordering system is room service [27], which allows patients to order a meal a la carte and receive it within 45 min. This model reduced food waste from 29% to 12%, compared to a traditional foodservice model. Finally, a two-portion size has been proposed to allow each patient to choose his or her desired quantity of food in a meal [16].

Starting from the results of our work, we suggested to the management of the three hospitals mainly two points of this list: establish an individual, simplified and flexible meal reservation process based on specific needs, preferences and nutritional choices; improve the quality of food on the basis of consumer satisfaction surveys. Indeed, one third of patients declared discarding food due to its low quality. Patient feedback is important in outcomes research and quality improvement efforts, as it provides a formal opportunity for feedback and demonstrates to patients that their opinions are valued by health professionals. Administrators and auditors of health care services are continuously seeking patient-reported outcomes to obtain indications of quality of care and the organisation of services.

However, applying these recommendations to the three hospitals involved in our study would be quite difficult because the foodservice is outsourced and therefore, beyond the control of hospital managers. At the moment, we do not know whether suppliers are interested in reducing food waste; they could have other conflicting objectives and interests. However, we wrote to the managers of the three hospitals and the foodservice suppliers to inform them of the results of our study and invite them to adopt whichever of the ministerial recommendations they consider most appropriate and easily applicable. In addition, our method has the advantage of integrating food waste data with other patient satisfaction information.

## 5. Limitations

Although our study has a large sample size and the results were homogeneous among the three hospitals, we cannot apply these findings to all the hospitals in the region. An innovative but critical point of our research was the methodology we used to calculate the food wasted in these facilities: we invited the patients to declare the amount of food they had discarded themselves. With this methodology, we could hypothesize two information biases: the recall bias and the interviewer bias. Due to the recall bias, any errors of assessment by patients could presumably go in both directions in terms of both over- and underestimation of wasted food, which would make our estimates fairly reliable. Instead the interviewer bias, i.e., the desire to please the interviewer, could have caused an underestimation of the discarded food. Further studies are needed to verify the reliability of this methodology and to compare it to the standard method in this field (i.e., weighing the food before it is served and the remainder discarded).

## 6. Conclusions 

Food waste is an important topic with a significant impact on environment, community and public health. Despite the efforts made to date, it requires more research, more public recognition and more political attention. An important step toward a reduction of food waste is Goal 12.3 of the UN Sustainable Development [28]. This goal targets, by 2030, a 50% reduction in food waste at the retail and consumer levels, in addition to a reduction of food losses along production and supply chains. Existing studies on food waste have also failed to investigate managerial attitudes and approaches to minimizing food waste. Future research should focus on management approaches to reducing food waste. Managing food waste in various geographical contexts offers a research opportunity. There is evidence to suggest that the cultural background of consumers can play a role in the production of food waste [29]. Other recent studies showed that in developed countries, food is wasted mainly in the final stage of the consumer supply chain, demonstrating how much consumer attitudes and behaviour influence the production of food waste [30]. This could allow the development of case-specific food waste prevention plans addressing both the material and socio-economic aspects of food waste production [31].

Our study describes food waste from the patient’s point of view in three hospitals in Campania region in Italy, analysing possible causes. Therefore, our findings represent a useful framework of the food discarded by patients in three great hospitals in this area. Our results show a worrying percentage of food waste requiring a resolute intervention by the hospital managers. The effects of this waste could have not only economic or environmental consequences, but also indirect consequences on the health of patients.

## Figures and Tables

**Table 1:** Calculation of total percentages of food wasted starting from a single percentage (first plate).

% of Food Wasted	% of Patients	Coefficient	% of Patients X Coefficient
100	16.0	1	16.0
75	11.0	0.75	8.3
50	23.4	0.50	11.7
25	10.1	0.25	2.5
0	39.5	0	0
Total % of food wasted			38.5

**Table 2:** Socio-demographic characteristics of the participants.

Characteristics		N	%
Gender	Female	443	58.1
	Male	319	41.9
	Total	762	100.0
Age	18–40	191	25.0
	41–60	193	25.3
	61–80	309	40.6
	80–100	69	9.1
	Total	762	100.0
Marital status	Unmarried	97	12.7
	Married	574	75.3
	Other	91	12.0
	Total	762	100.0
Education	≤Primary school	185	24.3
	Middle school	236	31.0
	High school	249	32.6
	Degree	92	12.1
	Total	762	100.0
Employment	Employed	545	71.5
	Unemployed	217	28.5
	Total	762	100.0
Nationality	Italian	728	95.5
	Not Italian	34	4.5
	Total	762	100.0

**Table 3:** Percentage of food waste by single dish according to the patients’ evaluation.

Dish	Percentage of Food Waste (%)					Total Food Waste (%) °
	100	75	50	25	0	%
First plate	16.0	11.0	23.4	10.1	39.5	38.5 (32.2–42.0) *
Second plate	15.7	13.1	23.8	9.2	38.2	39.7 (35.8–43.0)
Side plate	40.7	8.3	12.6	7.0	31.5	55.0 (50.1–56.7)
Fruit	25.1	3.7	12.5	4.3	54.5	35.2 (32.2–39.7)
Total food waste						41.6%

° see Table 1 for calculation. * Lowest and highest percentages of the three hospitals.

**Table 4:** Food waste disaggregated according to sociodemographic characteristics.

Sociodemographic Characteristics		Food Waste ≥ 50%	%	Food Waste < 50%	%	RR	Confidence Interval	Crude *p* Value	Adjusted *p* value °
Age	18–60	191	49.7	193	50.3	1			
	61–100	193	51.1	185	48.9	1.03	0.89–1.18	0.716	
Gender	Man	122	38.2	197	61.8	1			
	Woman	262	59.1	181	40.9	1.51	1.31–1.74	0.000	0.000
Education	Low *	210	49.9	211	50.1	1			
	High **	174	51.0	167	49.0	1.02	0.89–1.18	0.753	
Nationality	Foreign	12	35.3	22	64.7	1			
	Italian	372	51.1	356	48.9	1.45	0.91–2.29	0.072	0.206
Employment	Employed	257	47.2	288	52.8	1			
	Unemployed	127	58.5	90	41.5	1.27	1.07–1.52	0.005	0.705

° Multivariate logistic regression (the variables with a crude *p* value ≤ 0.25, gender, nationality and employment, have been included in the model). * Low includes ≤ primary school and middle school; ** High: high school and degree.

**Table 5:** Food wasted from the first plate according to the opinions of patients regarding food quality and foodservice.

Variables		Food Waste ≥ 50%	%	Food Waste < 50%	%	Total	%	RR	Confidence Interval	Crude *p* value	Adjusted *p* value ^+^
Importance	^ None	63	47.4	70	52.6	133	17.5	1			
placed on meal	^^ High	321	51.0	308	49.0	629	82.5	1.07	0.90–1.29	0.442	–
Food safety	Always	255	45.5	306	54.5	561	85.4	1			
	Never	66	68.8	30	31.3	96	14.6	1.51	1.29–1.78	0.000	0.372
Right	* Always	250	46.8	284	53.2	534	70.1	1			
Temperature	** Never	134	58.8	94	41.2	228	29.9	1.25	1.09–1.45	0.003	0.773
Food	° Good	229	42.7	307	57.3	536	70.4	1			
presentation	°° Poor	155	68.9	70	31.1	225	29.6	1.61	1.41–1.84	0.000	0.166
Food variety	° Good	244	45.8	289	54.2	533	69.9	1			
	°° Poor	140	61.1	89	38.9	229	30.1	1.33	1.16–1.53	0.000	0.668
Time food is	Always	231	49.0	240	51.0	471	61.8	1			
served	Never	153	52.6	138	47.4	291	38.2	1.07	0.92–1.25	0.343	–
Taste of food	Good	223	40.3	330	59.7	553	72.6	1			
	Poor	161	77.0	48	23.0	209	27.4	1.91	1.68–2.17	0.000	0.011
Satisfaction with	Good	243	41.8	338	58.2	581	76.2	1			
foodservice	Poor	141	77.9	40	22.1	181	23.8	1.86	1.64–2.10	0.000	0.018
Courtesy of	Always	345	49.1	358	50.9	703	92.3	1			
the staff	Never	35	64.8	19	35.3	54	7.1	1.32	1.07–1.63	0.026	0.797
Food brought in	Never	233	44.0	296	56.0	529	69.4	1			
from outside	Always	151	64.8	82	35.2	233	30.6	1.59	1.31–1.92	0.000	0.011

^+^ Multivariate logistic regression (the variables with a crude *p* value ≤ 0.25, gender, nationality and employment, have been included in the model). * Always includes: always and often. ** Never includes: sometimes, rarely, and never. ° Good includes: very good, good, and sufficient. °° Poor includes: poor and very poor. ^ None includes: none, little, and moderate. ^^ High includes: high and very high.

**Table 6:** Reasons for food waste.

Reasons	N	%
Poor quality	258	33.9
Different eating habits	164	21.5
Poor appetite	129	16.9
Other reason	116	15.3
Feeling full	43	5.6
No answer	52	6.8
Total	762	100

## Supplementary Materials

The following are available online at https://www.mdpi.com/1660-4601/16/22/4330/s1, File S1: Questionnaire.
