# Supplementary material for: Patient Evaluation of Food Waste in Three Hospitals in Southern Italy
Source: Int J Environ Res Public Health. 2019 Nov 6;16(22):4330. doi: 10.3390/ijerph16224330 (PMC6888165; doi:10.3390/ijerph16224330)
Supplement: Supplementary file 1 [file ijerph-16-04330-s001.pdf]

Hospital: __________ Date: __________

FOODSERVICE EVALUATION QUESTIONNAIRE

1) How would you rate the quality of food in this hospital?

1. Very poor
2. Poor
3. Sufficient
4. Good
5. Very good

2) How did you expect the quality of food before hospitalization to be?

1. Very poor
2. Poor
3. Sufficient
4. Good
5. Very good
6. I had no expectations

3) How would you rate the quality of food in this hospital in relation to your expectations for hospital food?

1. Much worse
2. Worse
3. Equal
4. Better
5. Much better
6. I had no expectations

4) What importance does it attach to mealtimes in the hospital?

1. None
2. Little
3. Enough
4. Much
5. Very much

5) Do you think that food is produced and distributed in a controlled and safe manner?

1. Never
2. Rarely
3. Sometimes
4. Often
5. Always

6) How would you rate the variety of menus offered?

1. Very poor
2. Poor
3. Sufficient
4. Good
5. Very good

7) How would you rate the quantity of portions proposed?

1. Very little
2. Poor
3. Sufficient
4. Abundant

5. Very abundant

8) How would you judge the presentation of food?

1. Very poor
2. Poor
3. Sufficient
4. Good
5. Very good

9) Do you think the food temperature is appropriate?

1. Never
2. Rarely
3. Sometimes
4. Often
5. Always

10) Do you think meal times are appropriate?

1. Yes
2. No

11) In which percentage did you consume your meals?

First plate

1. Nothing / almost nothing
2. About ¼
3. About half
4. About ¾
5. All / almost all

Second plate

1. Nothing / almost nothing
2. About ¼
3. About half
4. About ¾
5. All / almost everything

Side plate

1. Nothing / almost nothing
2. About ¼
3. About half
4. About ¾
5. All / almost everything

Fruit

1. Nothing / almost nothing
2. About ¼
3. About half
4. About ¾
5. All / almost everything

12) During hospitalization, did you bring in food from home □ or from another catering service □?

1. Never
2. Rarely
3. Sometimes
4. Often

5. Always

13) If you did it, in particular which one?

	YES	NO
1. First plate	□	□
2. Second plate	□	□
3. Side plate	□	□
4. Fruit	□	□

5. Other ______________

14) What do you think about the reason for not eating meals?

1. Low appetite
2. Feeling of satiety
3. Poor quality
4. Different eating habits
5. Other ___________

15) Do you think the staff who deliver the meals are friendly and polite?

1. Never
2. Rarely
3. Sometimes
4. Often
5. Always

16) How would you rate the overall foodservice?

1. Very poor
2. Poor
3. Sufficient
4. Good
5. Very good

17) How long have you been in hospital (this time)?

____

18) Department

____

19) Have you been in hospital before?

1. Yes
2. No

20) If you have already been admitted, where?

1. In this hospital
2. In a hospital in the same region
3. In a hospital from another region, which one? ____________
4. In a foreign hospital, such as?

21) If you have already been admitted, how would you rate the quality of the foodservice compared to your previous experience?

1. Much worse
2. Worse
3. Equal
4. Better
5. Much better

22) Sex?

1. M
2. F

23) Age?

____

24) Nationality?

____

25) Education:

1. Primary school
2. Middle school
3. High school
4. College degree
5. None

26. Civil status:

1. Single
2. Married
3. Separated/divorced
4. Other

27) Profession

____

28) In general, how would you rate the quality of received hospital care?

1. Very poor
2. Poor
3. Sufficient
4. Good
5. Very good
